# Acute acquired comitant esotropia related to excessive Smartphone use

**DOI:** 10.1186/s12886-016-0213-5

**Published:** 2016-04-09

**Authors:** Hyo Seok Lee, Sang Woo Park, Hwan Heo

**Affiliations:** Department of Ophthalmology, Chonnam National University Medical School and Hospital, 42 Jebong-ro, Dong-Gu, Gwang-Ju, 61469 South Korea

**Keywords:** Esotropia, Medial rectus recession, Smartphone, Video display terminal

## Abstract

**Background:**

To describe the clinical characteristics and outcomes of acute acquired comitant esotropia (AACE) related to excessive smartphone use in adolescents.

**Methods:**

The medical records of 12 patients with AACE and a history of excessive smartphone use were retrospectively reviewed, and the duration of smartphone use, angle of deviation, refractive error, stereopsis, and treatment options were analyzed.

**Results:**

All patients showed convergent and comitant esotropia ranging from 15 to 45 prism diopters (PD; average: 27.75 ± 11.47 PD) at far fixation. The angle of deviation was nearly equivalent for far and near fixation. Every patient used a smartphone for more than 4 h a day over a period of several months (minimum 4 months). Myopic refractive errors were detected in eight patients (average:−3.84 ± 1.68 diopters (D]), and the remaining four patients showed mild hyperopic refractive error (average: +0.84 ± 0.53 D). Reductions in esodeviation were noted in all patients after refraining from smartphone use, and bilateral medial rectus recession was performed in three patients with considerable remnant esodeviation. Postoperative exams showed orthophoria with good stereoacuity in these patients.

**Conclusion:**

Excessive smartphone use might influence AACE development in adolescents. Refraining from smartphone use can decrease the degree of esodeviation in these patients, and remnant deviation can be successfully managed with surgical correction.

## Background

Acute acquired comitant esotropia (AACE) is an unusual presentation of esotropia in older children and adults [1]. Its prevalence remains unknown, but it is generally considered rare [2]. Three main types have been defined and later modified by previous investigators: (1) Swan type: esotropia due to the disruption of fusion (precipitated by monocular occlusion or loss of vision in one eye); (2) Burian-Franceschetti type: esotropia characterized by minimal hypermetropia and diplopia, often associated with physical or psychological stress; and (3) Bielschowsky type: esotropia that occurs in adolescents and adults with varying degrees of myopia, and shows equal deviation at distance and near fixation [3, 4]. The mechanism of Bielschowsky type AACE is thought to be uncorrected myopia with excessive near work (holding printed materials or sewing excessively close to the eye), resulting in an inability to maintain balance between the converging and diverging forces of the eye, and the subsequent development of increased tonus of the medial rectus muscles, leading to esotropia [3]. Other rare types of AACE have also been reported, such as refractive-accommodative type AACE, and AACE associated with accommodative spasm or intracranial diseases [2, 5, 6].

The adoption of mobile technologies and wireless communication infrastructure is a global phenomenon [7, 8]. Among the existing technologies, smartphones have been one of the most prominent success stories of the last decade. In a relatively short period of time, smart mobile technology has significantly penetrated society in the Western world and globally, including South Korea. Since the introduction of the iPhone 3GS (Apple Inc., Cupertino, CA, USA) in South Korea in November 2009, smartphone distribution has dramatically increased and has become popular in a short period of time owing to South Korea’s advanced information technology development. According to a report from the Korea Communications Commission, the number of smartphone users was estimated to be over 35 million in July, 2013 [9].

More specifically, smartphone possession has become surprisingly popular among adolescents and young adults. In 2010, only 5.8 % of adolescents in South Korea owned smartphones, but this number strikingly increased to 36.2 % in 2011, and exploded by nearly 15-fold to 81.5 % in 2013 [10]. However, the rapid spread of smartphones in today’s society is associated with potentially serious social problems, such as smartphone addiction. According to a survey about smartphone addiction conducted by the National Information Society Agency in 2012, the incidence was 8.4 % in South Korea, which was higher than the average internet addiction rate of 7.7 % [9, 11]. Moreover, teenagers and individuals in their twenties showed higher addiction rates than those in their thirties and forties, thereby illustrating the vulnerability of adolescents to smartphone addiction [12]. Addictive tendencies toward smartphones in adolescence are not confined to South Korea alone, but have rapidly become a significant mental health problem in other nations [13]. Since smartphones are closely connected to most of our daily life activities—functioning as a mobile phone, portable computer with internet access, and mp3 or video player—and many people spend a considerable amount of time fumbling with their smartphones, smartphone use can be regarded as representative near work in today’s contemporary era, replacing “classic” near work: reading or sewing. Thus, it is conceivable that smartphone addiction is a major contributor to excessive near work.

In this retrospective case series, we aimed to review our experience with AACE associated with excessive smartphone use, including its etiologies and outcomes.

## Methods

The medical records of all adolescents with AACE who were examined at the Pediatric Ophthalmology and Strabismus Service of Chonnam National University Hospital, between January 2009 and June 2014 were reviewed. Patients who met the following criteria were included in this retrospective study: (1) age of onset after 1 year of age; (2) age ≤16 years; (3) acute onset of comitant strabismus (same deviation in all gaze directions); (4) photographic evidence of absence of strabismus before esotropia onset; (5) corrected visual acuity of 20/20 in both eyes; and (6) a minimum follow-up period of 3 months [14]. Patients with a history of eye problems, including strabismus and amblyopia, previous ocular surgery, ophthalmic eye drop use, systemic diseases (including diabetes mellitus), or head trauma, were excluded. In total, 19 patients fulfilled the inclusion criteria. Among these, three patients were diagnosed with Burian-Franceschetti type AACE; one patient was diagnosed with Bielschowsky type AACE; and three patients were diagnosed with AACE related to neurologic disease. However, the other 12 patients did not meet the diagnostic characteristics for the three main types of AACE or AACE related to neurologic disease. Following extensive history taking and examination, the remaining 12 patients were identified as excessive smartphone users (smartphone use more than 4 h a day for more than four consecutive months, based on the statements of the patients and their parents) [9, 11]. This tendency was not found in the other seven patients, who either did not have a smartphone or used a smartphone for less than 2 h a day. Interestingly, the AACE patients presented to our clinic after the beginning of 2012, which corresponds with the period of rapid distribution of smartphones in South Korea. Therefore, we decided to analyze the clinical characteristics of the AACE patients who did not fit into the preexisting AACE classification. The following data were abstracted from the medical records: age, gender, presence of diplopia, visual acuity, duration of smartphone use, angle of deviation for near and distance fixation, manifest refractive error, cycloplegic refractive error, measurement of near stereoacuity with the Titmus test, Bagolini striated glasses test results, medical and surgical treatment, and recurrence. The alternate prism cover test was performed to measure the angle of deviation at 6 m and 33 cm fixation, as well as for all gaze directions with refractive correction. The manifest refractive error was measured with an automated refractometer (KR8900, Topcon Corp., Tokyo, Japan). Cycloplegic autorefraction was performed by a single investigator (HSL). To achieve adequate cycloplegia, 1 % atropine sulfate eye drop was instilled three times a day for 3 days by the parents or guardians at home, prior to visiting the clinic. On the 4th day, cycloplegia was assessed by pupil diameter ≥6 mm and no reaction to light or the accommodative target. Refractive error measurements were obtained by using an automated refractometer (KR8900, Topcon Corp.); five readings with a maximum 0.25 diopter (D) difference were recorded and averaged. The manifest and cycloplegic spherical equivalents of refractive error were calculated by using the algebraic sum of the dioptric powers of the sphere and half of the cylinder. All patients in this study underwent a complete neurological examination, including brain magnetic resonance imaging, conducted by a pediatric neurologist; the examinations revealed normal results in all patients except for the three who were diagnosed with AACE related to neurologic disease. Approval for this study was obtained from the Institutional Review Board of Chonnam National University Hospital (IRB No.: CNUH-2014-189), and the study adhered to the tenets of the Declaration of Helsinki. Written informed consent was given by the participants and their caregivers (legal guardian) for their clinical records to be used in this study. SPSS version 18.0 (SPSS Institute Inc., Chicago, IL, USA) was used for statistical analyses. The Wilcoxon signed rank test was used to compare the angle of deviation before and after smartphone restriction. *P* values < 0.05 were considered statistically significant.

## Results

The mean age of the 12 patients was 13.33 ± 3.31 years (range, 7–16 years; Table 1A and B), seven of which were female. The onset of esotropia preceded presentation at our clinic by an average of 5.83 ± 2.89 months (range, 2–10 months). Results of neurological examination as well as brain magnetic resonance imaging were normal. The average duration of smartphone use per day was 6.08 ± 1.78 h. The average duration of smartphone use prior to the eye examination was 10.5 ± 5.13 months. All patients stated that they usually viewed smartphones at a close reading distance (<30 cm). Nine patients complained of horizontal diplopia, but five patients stated that diplopia happened only intermittently, usually at distance fixation. None of the patients complained that the diplopia severely interfered with their daily lives.

**Table 1:** Clinical characteristics of patients with acute acquired comitant esotropia

A						
Patient Number	1	2	3	4	5	6
Age (years)/Sex	13/F	16/M	16/M	12/F	7/M	15/F
Wearing spectacles	−	+	+	+	−	+
Duration of esotropia (months)	3	8	8	10	10	5
Smartphone use per day (hours)	5	8	4	6	4	8
Duration of smartphone use (months)	6	12	10	12	12	8
Presence of diplopia	−	+	+	+	−	+
Presenting VA, Rt/Lt (logMAR)	0.0/0.0	0.0/0.0	0.0/0.0	0.0/0.0	0.0/0.0	0.0/0.0
UCVA, Rt/Lt (logMAR)	0.0/0.0	0.5/0.5	0.4/0.3	0.8/0.8	0.0/0.0	0.5/0.4
BCVA, Rt/Lt (logMAR)		0.0/0.0	0.0/0.0	0.0/0.0		0.0/0.0
Manifest refraction (SE), Rt/Lt (D)	+0.25/ +0.25	−3.00/ −3.50	−4.00/ −3.75	−4.50/ −4.50	−0.75/ −0.5	−4.50/ −4.25
Cycloplegic refraction (SE), Rt/Lt (D)	+1.50/ +1.50	−2.00/ −2.25	−3.50/ −3.50	−4.00/ −4.25	+0.25/ +0.25	−4.50/ −4.25
Spectacle glasses power (SE), Rt/Lt (D)	Nil	−3.00/ −3.50	−3.75/ −3.50	−4.00/ −4.25	Nil	−4.25/ −4.00
Sensory function (arc seconds) at initial presentation	Nil	Nil	Nil	Nil	Nil	Nil
Bagolini striated glasses test results	Unsuppressed esotropia	Unsuppressed esotropia	Unsuppressed esotropia	Unsuppressed esotropia	Unsuppressed esotropia	Unsuppressed esotropia
Esodeviation at initial presentation, distance/near (PD)	45/45	35/35	45/45	35/35	25/25	20/20
Esodeviation after restriction of smartphone use, distance/near (PD)	35/35	30/30	30/30	15/15	8/12	12/4
Operation	BMR	BMR	BMR	–	–	–
Esodeviation at last follow up visit, distance/near (PD)	Ortho/Ortho	Ortho/Ortho	Ortho/Ortho	15/15	8/12	12/6
Sensory function at last follow up visit (arc seconds)	40	40	100	100	100	40
Bagolini striated glasses tset results after operation	Normal binocular single vision	Normal binocular single vision	Normal binocular single vision	Nil	Nil	Nil
Recurrence	–	–	–	–	–	–
Follow-up period (months)	14	12	8	11	4	6
B						
Patient Number	7	8	9	10	11	12
Age (years)/Sex	15/F	16/M	15/F	7/M	16/F	12/F
Wearing spectacles	+	+	−	−	+	+
Duration of esotropia (months)	4	8	2	3	6	3
Smartphone use per day (hours)	8	5	5	4	8	8
Duration of smartphone use (months)	6	12	24	12	8	4
Presence of diplopia	+	+	+	−	+	+
Presenting VA, Rt/Lt (logMAR)	0.0/0.0	0.0/0.0	0.0/0.0	0.0/0.0	0.0/0.0	0.0/0.0
UCVA, Rt/Lt (logMAR)	1.0/1.0	1.0/1.0	0.0/0.0	0.0/0.0	0.8/0.8	0.4/0.4
BCVA, Rt/Lt (logMAR)	0.0	0.0		0.0/0.0	0.0/0.0	0.0/0.0
Manifest refraction (SE), Rt/Lt (D)	−4.25/ −4.75	−4.50/ −4.75	+0.50/ +0.25	+0.75/ +1.125	−7.5/ −7.5	−1.625/ −1.625
Cycloplegic refraction (SE), Rt/Lt (D)	−4.125/ −4.25	−3.50/ −4.00	+0.50/ +0.50	+1.0/ +1.25	−7.25/ −7.25	−1.5/ −1.375
Spectacle glasses power (SE), Rt/Lt (D)	−4.00/ −4.50	−4.00/ −4.375	Nil	Nil	−7.0/ −7.0	−1.50/ −1.375
Sensory function (arc seconds) at initial presentation	Nil	100	Nil	800	100	400
Bagolini striated glasses test results	Unsuppressed esotropia	Unsuppressed esotropia	Unsuppressed esotropia	Unsuppressed esotropia	Unsuppressed esotropia	Unsuppressed esotropia
Esodeviation at initial presentation, distance/near (PD)	25/30	15/15	35/35	20/20	8/10	25/25
Esodeviation after restriction of smartphone use, distance/near (PD)	20/20	15/12	25/25	10/10	6/0	15/15
Operation	–	–	–	–	–	–
Esodeviation at last follow up visit, distance/near (PD)	20/20	15/12	25/25	10/10	6/0	15/15
Sensory function at last follow up visit (arc seconds)	Nil	100	Nil	100	60	100
Recurrence	–	–	–	–	–	–
Follow up period (months)	3	3	3	3	6	6

*Abbreviations*: *VA* visual acuity, *UCVA* uncorrected visual acuity, *BCVA* best corrected visual acuity, *Rt* right, *Lt* left, *PD* prism diopter, *SE* spherical equivalent, *D* diopter, *BMR* bilateral medial rectus recession, *Ortho* Orthophoria

The mean manifest refractive errors were−2.81 ± 2.13 D in the right eyes and−2.88 ± 2.13 D in the left eyes. After cycloplegia, myopic refractive errors were detected in eight patients (average:−3.80 ± 1.74 D in the right eyes and−3.89 ± 1.73 D in the left eyes), and the remaining four patients showed mild hyperopic refractive error (average: +0.81 ± 0.55 D in the right eyes and +0.88 ± 0.60 D in the left eyes). Each patient presented with a visual acuity of 20/20 in both eyes on their first visit to our clinic (patients 1, 5, 9, and 10 had an uncorrected visual acuity of 20/20 in both eyes and all other patients showed visual acuity of 20/20 with their glasses). The esodeviations at initial presentation were comitant, ranging from 15 to 45 prism diopters (PD) at far (mean, 27.75 ± 11.47 PD) with full correction of refractive errors. In each patient, the angles of esodeviation were nearly equivalent for distance and for near fixation (differing by ≤ ±5 PD; mean, 28.33 ± 11.15 PD). After refraining from smartphone use for 1 month, all patients noted esodeviation improvement (17.50 ± 6.45 PD at far fixation [*p* = 0.003]; 17.13 ± 6.24 PD at near fixation [*p* = 0.002]) Slit lamp and fundoscopic examination revealed normal results in all patients. There were no apparent gaze limitations in either eye of any patient.

Strabismus surgery was advised for five patients who showed a small decrease in esodeviation after smartphone restriction and/or considerable remnant esodeviation (>15 PD). Three of these patients underwent bilateral medial rectus recession appropriate for the degree of esotropia under general anesthesia, whereas the other patients (7 and 9) refused surgical treatment. Postoperatively, all patients achieved orthophoria. The initial and final stereoacuity test results, and surgical interventions used, along with other parameters are presented in Table 1A and B.

## Discussion

Both similarities and differences exist between Bielschowsky type AACE (described originally by Bielschowsky and later modified by Hoyt and Good), and the patients described in our case series [3, 4]. Comitant esodeviation without evidence of paralysis of the extraocular muscles, similar deviation at distance and near fixation, as well as various degrees of myopia, are consistent with the definition suggested by Bielschowsky, Hoyt and Good. Further, we assumed that dynamic preponderance of the medial rectus muscles after sustained near work played a pivotal role in the development of esotropia in our patients. However, unlike Bielschowsky’s postulation in which uncorrected myopia was the key etiology of this form of strabismus, eight of 12 patients with myopic refractive error in our study wore glasses, and none of these patients was reluctant to wear glasses before or after presentation to our clinic. In addition, the corrected visual acuity of the eight patients with myopia was 20/20 in both eyes, and the uncorrected visual acuity was 20/20 in both eyes in the other four patients with hyperopia,

The Bagolini striated glasses test was performed in all patients at initial presentation, and the results showed unsuppressed esotropia in all cases [15]. We repeated the Bagolini striated glasses test in patients who underwent strabismus surgery at 3 months after the operation, and the results revealed normal binocular single vision in all three patients. And all patients who received strabismus surgery regained normal stereopsis proved by Titmus test. Moreover, mere smartphone restriction for 1 month led to a significant decrease in esodeviation in all patients, which would not occur in decompensated monofixation syndrome. Based on these observations, we believe that monofixation syndrome can be excluded in our patients [16].

Myasthenia gravis may present with acute comitant strabismus. However, our patients did not show any sign of ptosis, change in strabismus pattern, or other signs of muscle weakness throughout the follow-up period; further, the angle of deviation did not vary on repeated testing from day to day throughout the follow-up period. Although spasm of near reflex or accommodation is a possible differential diagnosis, this can be excluded based on the absence of episodic miosis, psychogenic or organic causes, consistency of eye deviation and refractive error in our patients after cycloplegia [17, 18]. Refractive esotropia is also a possible differential diagnosis, but all patients in our study were myopic or mildly hyperopic, and refractive correction did not show any change in the degree of esodeviation in patients with hyperopia. Bilateral sixth nerve paresis might evolve into comitant esodeviation without motility limitations in the future. However, the absence of trauma history, brain lesions, or vascular disorder, the similarity in esodeviation at near and far fixation, and the reduction in esodeviation after smartphone restriction could be discriminating factors between bilateral sixth nerve paresis and comitant esodeviation in our patients.

As proposed in previous articles, many authors have emphasized that a high index of clinical suspicion should be maintained for intracranial lesions as a cause of AACE, because AACE can be the sole sign of intracranial disease [19–21]. However, all patients in our case series had undergone neurologic examination, including neuroimaging, and no abnormalities were detected.

Burian-Franceschetti type AACE can also be a possible diagnosis. Comitant convergent strabismus without a definitive underlying cause, the presence of diplopia, the moderate degree of the angle of deviation, and good functional outcome after strabismus surgery meets the diagnostic criteria. However, most of our patients were myopic and none of our patients had experienced recent physical or psychological stress due to exhaustion that might have caused acute onset of eyeball deviation in the aforementioned type of esotropia.

The type of esotropia observed in our case series most closely resembles “acute concomitant esotropia of adulthood” described by Spierer [22]. They share common characteristics, such as the development of comitant esotropia with normal corrected visual acuity in both eyes, regaining of normal stereopsis after surgical correction of esotropia, as well as similar angle of deviation at distance and near fixation, absence of neurologic disease, and mainly myopic refraction error. The mechanism by which patients with myopia and good stereopsis develop comitant esotropia is still not clear.

Video display terminal (VDT) work itself was shown to induce abnormalities in accommodation and vergence compared with ordinary hard copy work (non-VDT work) [23, 24]. Thus, it is conceivable that excessive smartphone use at a close reading distance and the resultant abnormalities in accommodation and vergence in adolescents with low fusional divergence capacity or previous latent esophoria (inherently susceptible to the development of esotropia) can lead to dynamic preponderance of the medial rectus muscles, resulting in the development of manifest esotropia; the mechanism of which was initially proposed by Bielschowsky [3, 4, 25].

The absence of diplopia in some patients and the presence of only intermittent diplopia at distance fixation in others might indicate that dynamic hypertonus of the medial rectus muscles and the resultant development of esotropia progress slowly [26]. Refraining from smartphone use caused a decrease in the degree of esodeviation, which partially supports this mechanism. However, evidence is insufficient to supportthis proposal. The exact mechanism by which comitant esotropia develops in myopic or slightly hyperopic patients without previous manifest deviation remains unclear and has yet to be determined.

The orthophoric position and normal fusional capacity were re-established postoperatively in three patients who received bilateral medial rectus recession. As Spierer [22] proposed, normally developed binocular vision is disrupted after the onset of strabismus; therefore, the binocular capacity is potentially preserved and is later regained after the strabismus is surgically corrected.

The limitations of this case series include small sample size and the fact that we studied only patients with AACE who used smartphones excessively. AACE is a disease entity with undefined prevalence, but it is certainly considered rare. Therefore, although medical chart review was performed for patients who had visited the tertiary pediatric ophthalmology and strabismus service during a period of several years, the sample size was small [14]. In addition, we could not perform a comparative analysis between AACE patients with and without history of excessive smartphone use because of the paucity of AACE patients.

A distinct causal relationship between AACE and excessive smartphone use was not proven in our patients. However, other types of AACE, such as Bielschowsky type, Burian-Franceschetti type, AACE caused by intracranial tumor, and other causes of esotropia in adolescences, can be excluded in our case series. Further, extensive and comprehensive questioning and history taking revealed that only excessive smartphone use (more than 4 h per day) for a long period of time was common in all patients with AACE who were not diagnosed with Bielschowsky type, Burian-Franceschetti type, and AACE related to neurologic disease. In addition, smartphone restriction led to a significant decrease in esodeviation in all patients. Therefore, we speculate that excessive smartphone use could lead to the development of AACE.

Further, it is unclear whether other confounding factors (e.g., other forms of near work such as reading or sewing) may have influenced the clinical results. However, according to questioning and history taking, a gross difference did not exist in hours spent studying, reading, or other possible near-visual activities in AACE patients with a history of excessive smartphone use, when compared with AACE patients due to other causes. Further case-controlled studies with larger study populations are warranted. Moreover, we did not obtain an accommodation measurement by using dynamic retinoscopy or other devices that would give a more objective measure of accommodative power.

## Conclusion

In conclusion, excessive smartphone use at a close reading distance might influence the development of rarely occurring AACE in patients with myopia or mild hyperopia, good corrected visual acuity, and binocularity. AACE can potentially be induced by increased tonus of the medial rectus muscles resulting from the sustained near work itself, and disrupted accommodation and vergence by VDT work. In these cases, refraining from smartphone use can decrease the amount of esodeviation, and successful management of residual esotropia and restoration of binocularity can be achieved with bilateral medial rectus recession. Further studies with larger sample sizes and long-term follow-up periods are warranted.

## Availability of data and materials

All data sets on which the conclusions of our article rely is presented in the article as a table.

## Abbreviations

AACE: acute acquired comitant esotropia
D: diopters
PD: prism diopters
VDT: video display terminal
